# Deciphering Unexpected Vascular Locations of *Scedosporium* spp. and *Lomentospora prolificans* Fungal Infections, France

**DOI:** 10.3201/eid3006.231409

**Published:** 2024-06

**Authors:** Carole Vignals, Joseph Emmerich, Hugues Begueret, Dea Garcia-Hermoso, Guillaume Martin-Blondel, Adela Angoulvant, Damien Blez, Patrick Bruneval, Sophie Cassaing, Emilie Catherinot, Pierre Cahen, Cécile Moluçon-Chabrot, Carole Chevenet, Laurence Delhaes, Lélia Escaut, Marie Faruch, Frédéric Grenouillet, Fabrice Larosa, Lucie Limousin, Elisabeth Longchampt, François Mellot, Céline Nourrisson, Marie-Elisabeth Bougnoux, Olivier Lortholary, Antoine Roux, Laura Rozenblum, Mathilde Puges, Fanny Lanternier, Didier Bronnimann

**Affiliations:** Centre Hospitalier Universitaire de Bordeaux, Bordeaux, France (C. Vignals, H. Begueret, L. Delhaes, M. Puges, D. Bronnimann);; Hôpital Saint-Joseph, Paris, France (J. Emmerich);; Institut Pasteur, Paris (D. Garcia-Hermoso, M.-E. Bougnoux, O. Lortholary, F. Lanternier);; Centre Hospitalier Universitaire de Toulouse, Toulouse, France (G. Martin-Blondel, S. Cassaing, M. Faruch);; Hôpital Bicêtre, Le Kremlin-Bicêtre, France (A. Angoulvant, L. Escaut);; Hôpital Européen Georges Pompidou, Paris (D. Blez, P. Bruneval);; Hôpital Foch, Suresnes, France (E. Catherinot, P. Cahen, L. Limousin, E. Longchampt, F. Mellot, A. Roux);; Centre hospitalier universitaire de Clermont-Ferrand, Clermont-Ferrand, France (C. Moluçon-Chabrot, C. Chevenet, C. Nourrisson);; Centre hospitalier universitaire de Besançon, Besançon, France (F. Grenouillet);; Centre Hospitalier Universitaire de Dijon, Dijon, France (F. Larosa);; Hôpital Necker-enfants malades AP-HP, Paris (M.-E. Bougnoux, O. Lortholary, F. Lanternier);; Hôpital Pitié-Salpêtrière AP-HP, Paris (L. Rozenblum)

**Keywords:** *Scedosporium*, *Lomentospora*, *Lomentospora prolificans*, scedosporiosis, lomentosporiosis, arteritis, aortitis, mycotic aneurysm, vascular infections, fungi, France

## Abstract

*Scedosporium* spp. and *Lomentospora prolificans* are emerging non-*Aspergillus* filamentous fungi. The Scedosporiosis/lomentosporiosis Observational Study we previously conducted reported frequent fungal vascular involvement, including aortitis and peripheral arteritis. For this article, we reviewed 7 cases of *Scedosporium* spp. and *L. prolificans* arteritis from the Scedosporiosis/lomentosporiosis Observational Study and 13 cases from published literature. Underlying immunosuppression was reported in 70% (14/20) of case-patients, mainly those who had solid organ transplants (10/14). Osteoarticular localization of infection was observed in 50% (10/20) of cases; infections were frequently (7/10) contiguous with vascular infection sites. *Scedosporium* spp./*Lomentospora prolificans* infections were diagnosed in 9 of 20 patients ≈3 months after completing treatment for nonvascular scedosporiosis/lomentosporiosis. Aneurysms were found in 8/11 aortitis and 6/10 peripheral arteritis cases. Invasive fungal disease­–related deaths were high (12/18 [67%]). The vascular tropism of *Scedosporium* spp. and *L. prolificans* indicates vascular imaging, such as computed tomography angiography, is needed to manage infections, especially for osteoarticular locations.

*Scedosporium* spp. and *Lomentospora prolificans* are non-*Aspergillus* filamentous fungi causing increasingly recognized invasive fungal disease (IFD) in both immunocompromised and immunocompetent patients (*1*,*2*). *Scedosporium* spp. comprise *S. apiospermum* complex species, which includes *S. apiospermum sensu stricto* and *S. boydii*; *L. prolificans* was previously known as *S. prolificans* (*3*). In immunocompetent patients, localized infections have been described, such as mycetomas, osteoarticular infections (*4*), or central nervous system (CNS) infections after near-drownings. In immunocompromised hosts, scedosporiosis and lomentosporiosis mainly affect the lungs and CNS or are disseminated (*5*–*11*). We previously conducted the Scedosporiosis/lomentosporiosis Observational Study (SOS) and reported non-CNS vascular involvement (aorta or peripheral arteries excluding CNS arteries) in 24% of disseminated infections (*12*). Although CNS vascular involvement during invasive scedosporiosis has been described, a scedosporiosis/lomentosporiosis case series of non-CNS vascular infections has not been reported (*5*–*11*,*13*–*17*). We describe clinical, imaging, and detailed histopathologic characteristics of all patients with non-CNS vascular scedosporiosis/lomentosporiosis infections identified in the SOS and review cases reported in published literature. 

## Methods

### Ethics Approval

The research was approved by the Institut Pasteur Internal Review Board (approval no. 2009–34/IRB) and by the Commission Nationale de l’Informatique et des Libertés in accordance with the laws of France. All clinical data were recorded anonymously.

### Study Design

The SOS included all retrospective cases of proven and probable invasive scedosporiosis/lomentosporiosis (IS) characterized at the National Reference Center for Invasive Mycoses and Antifungals, France, during January 2005–March 2017 (*12*). We performed a medical records review of all patients in the SOS who had non-CNS vascular infections (aortitis or peripheral arteritis) caused by *Scedosporium* spp. or *L. prolificans*. In addition, we performed an electronic literature search for case reports in PubMed on October 10, 2021, by using the following search filter: (Scedospori*[Title/Abstract] OR Pseudallescheri*[Title/Abstract] OR Lomentospori*[Title/Abstract]) AND (invasive[Title/Abstract] OR disseminated[Title/Abstract] OR infection[Title/Abstract]) AND (case[Title/Abstract] OR patient[Title/Abstract] OR report[Title/Abstract]). We selected all articles in English, French, or Spanish that reported proven or probable non-CNS vascular infections caused by *Scedosporium* spp., including *Pseudallescheria* spp., or *L. prolificans*, including *S. prolificans* and *S. inflatum*. We checked reference lists of selected articles for other relevant studies. We only included published case reports or case series with detailed documentation of clinical history, diagnostic methods, treatment, and outcomes. Data from 1 SOS case (patient 5) had been published previously, and we excluded this case from the literature review.

For each case, we reported demographic conditions, underlying diseases/conditions, vascular impairment sites, signs and symptoms, other infected sites, microbiologic results, antifungal treatments, surgical therapy, and clinical outcomes by using a standard anonymous case report form. Each SOS case was reviewed by 4 investigators (C.V., J.E., F.L., and D.B.); each literature review case was reviewed by 2 investigators (C.V. and D.B.).

### Definitions

We defined non-CNS vascular infection as either imaging of structural abnormalities of the arterial wall suggestive of aortitis/arteritis (*18*) associated with a concomitant IS diagnosis (Appendix), a positive culture from an intraoperative arterial wall sample, or anatomopathologic evidence of arterial wall fungal infection with a concomitant IS diagnosis. We excluded cases of arterial thrombosis without evidence of parietal impairment. We defined proven and probable IS according to the 2019 European Organization for Research and Treatment of Cancer/Mycoses Study Group criteria (*19*), modified by including trauma and near-drowning as risk factors.

We considered 6 main underlying conditions for IFD, but only 1 condition was assigned to each patient as follows: malignancies; solid organ transplantation; systemic inflammatory disease; contamination by traumatic penetration, injury, surgery, or near-drowning (trauma/inoculation); other, for miscellaneous underlying medical conditions, including neutropenia, chronic renal or respiratory insufficiency, chronic respiratory disease, diabetes mellitus, HIV infection, or corticosteroids administration; and no risk factor in the absence of all previous factors.

We considered infections to be disseminated when they involved >2 noncontiguous sites or were associated with fungemia. We defined breakthrough infections as those occurring in patients who received antifungal therapy for >7 days within 30 days before IS diagnosis and defined prior colonization as a *Scedosporium/Lomentospora* culture within a nonsterile clinical sample without signs of infection before IS diagnosis. We defined follow-up as the period from IS diagnosis to last patient contact or death if vascular infection was diagnosed at the time of IS diagnosis or within 3 months of diagnosis; otherwise, we defined it as the period from vascular infection diagnosis to last patient contact or death. We considered IFD-related death as death supposedly caused by IFD. We defined radical surgery as complete resection of the vascular infection site, including vascular prothesis removal, whether or not it was associated with vascular reconstruction. For analyses, we described quantitative variables as medians with interquartile ranges and categorical variables as numbers and percentages.

## Results

### SOS Case-Patients

**Figure 1:**
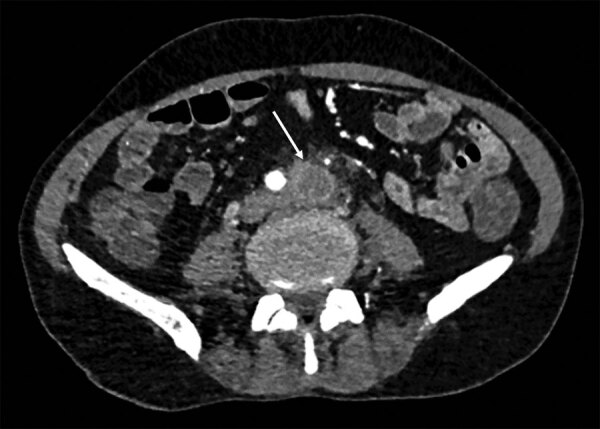
Abdominal computed tomography scan for case-patient 1 showing arteritis in study of unexpected vascular locations of *Scedosporium* spp. and *Lomentospora prolificans* fungal infections, France. Arrow indicates primitive left iliac artery thrombosis and perivascular soft tissue contrast. Data are from the Scedosporiosis/lomentosporiosis Observational Study (*12*).

Case-patient 1 was a 52-year-old man who had liver and kidney transplants because of alcoholic cirrhosis and IgA nephropathy. He had dermohypodermitis of the left lower limb caused by *S. apiospermum* and was treated with voriconazole for 18 months. Despite initial clinical success, *S. apiospermum* oligoarthritis developed 3 months after the patient stopped antifungal therapy; infection was documented in cultures of left knee joint liquid. He received a combination of voriconazole, caspofungin, and terbinafine and reduction of his immunosuppressive regimen. No evidence of endocarditis was found on an echocardiogram. Blood culture results were negative. Because fever persisted during treatment, positron emission tomography (PET)/computed tomography (CT) and abdominal/pelvic CT were performed, which identified multiple osteoarticular localizations (ankles, knees, left sacroiliac joint, left hip, T8 spondylitis, L5–S1 spondylodiscitis), left primitive iliac arteritis (thrombosis with perivascular and soft tissue contrast) (Figure 1) and abdominal bifurcation aortitis (perivascular and soft tissue contrast associated with focal hypermetabolism). The patient died 2 months later from multiorgan failure accompanied by acute left lower limb ischemia and possible mesenteric ischemia.

**Figure 2:**
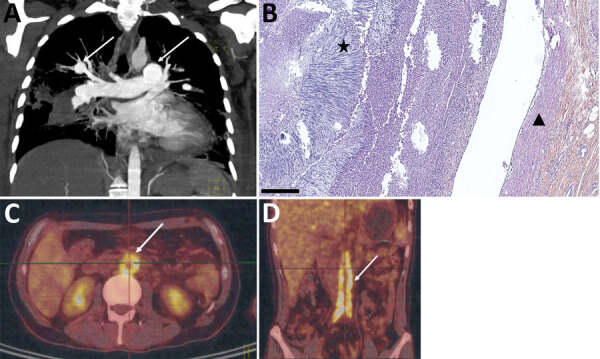
Imaging of pulmonary arteritis and abdominal aortitis for case-patients 2 and 3 in study of unexpected vascular locations of *Scedosporium* spp. and *Lomentospora prolificans* fungal infections, France. A) Thoracic computed tomography scan for case-patient 2. Arrows indicate left pulmonary artery and right lobar pulmonary artery mycotic aneurysms. B) Hematoxylin-eosin-saffron stain of lung tissue from postmortem analysis of case-patient 2. Triangle indicates bronchial artery wall; star indicates bronchial artery thrombus consisting of radially-disposed multiple septate hyphae. Scale bar indicates 250 μm. C, D) Positron emission tomography-computed tomography scans of case-patient 3. C) Arrow indicates intense abdominal aorta hypermetabolism. D) Arrow indicates abdominal aorta and primitive iliac artery hypermetabolisms. Data are from the Scedosporiosis/lomentosporiosis Observational Study (*12*).

Case-patient 2 was a 31-year-old woman recovering from a severe road accident in muddy water. She was hospitalized 2 months after the accident for pneumonia and bilateral pulmonary embolisms. She was treated empirically with antimicrobial drugs and anticoagulation therapy, which was stopped a few days later because of massive hemoptysis. She experienced persistent fever and epileptic seizures; an echocardiogram and CT scan revealed tricuspid endocarditis associated with cerebral abscess and bilateral aneurysms of pulmonary arteries rapidly growing and partially thrombosed (Figure 2, panel A). At that time, *S. apiospermum* was identified from blood cultures. She received long-term therapy with voriconazole and caspofungin, which was successful. However, chronic pulmonary arterial hypertension developed, requiring cardiopulmonary transplantation while she was undergoing antifungal treatment for >2 years. She died the day of transplantation because of a pulmonary hilum twist. Postmortem examination showed highly altered pulmonary arteries with major intimal fibrosis and a thrombus containing multiple fungal septate hyphae (Figure 2, panel B).

Case-patient 3 was a 32-year-old man who had macrophage activation syndrome, acute renal failure requiring hemodialysis, and multiple necrotic cutaneous lesions after acute cocaine intoxication. Febrile acute shoulder arthritis and sternal osteochondritis developed for which he underwent surgical debridement; *S. apiospermum* was identified in perioperative shoulder samples, and *L. prolificans* was identified in sternum samples. Thoracic-abdominal-pelvic CT and PET/CT scans revealed pseudonodular pulmonary lesions, abdominal aortitis and primitive iliac arteritis with perivascular contrasts, arterial wall thickenings, and intense vascular hypermetabolism (Figure 2, panels C, D). He was successfully treated with voriconazole, terbinafine, and miltefosine for 3 years, along with γ-interferon in the absence of initial improvement. Follow-up imaging performed 2.5 years after treatment initiation found a clear reduction of periaortic inflammatory thickening and vascular hypermetabolism. Antifungal treatment was stopped after 3 years without any clinical sign of relapse 1 year after interruption.

**Figure 3:**
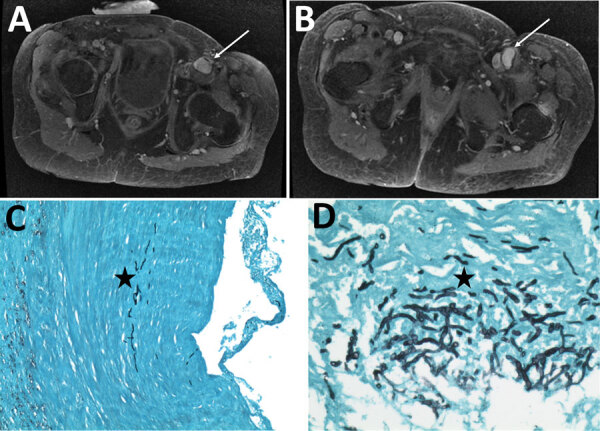
Radiologic and histopathologic analysis of arteritis in case-patient 4 in study of unexpected vascular locations of *Scedosporium* spp. and *Lomentospora prolificans* fungal infections, France. A, B) Pelvic magnetic resonance imaging with T1 gadolinium contrast showing femoral arteritis. A) Arrow shows aneurysms involving the left common femoral artery with posterior mural thrombus. B) Arrow shows left common femoral artery bifurcation. C, D) Grocott-Gomori methenamine silver stain of femoral artery section. C) Star indicates septate fungal hyphae invading the iliofemoral artery tunica media. Original magnification ×100. D) Star indicates septate fungal hyphae invading the iliofemoral artery tunica media. Original magnification ×400. Data are from the Scedosporiosis/lomentosporiosis Observational Study (*12*).

Case-patient 4 was a 51-year-old woman who had focal segment glomerulosclerosis requiring a kidney transplant, which was complicated by acute graft rejection. Closing surgery of her arteriovenous femoral fistula was complicated by a *Pseudomonas aeruginosa* infection at the surgical site, which was treated by a femoral bypass graft and antimicrobial drugs. Postoperatively, a femoral graft thrombosis and perivascular collection developed and was treated by graft removal, and a 3-month voriconazole course was begun because of positive results for *S. apiospermum* from a superficial swab culture. Eight months later, new onset of fever and left limb pain led to discovery of 2 mycotic aneurysms in the left common femoral artery (Figure 3, panels A, B), multiple left thigh abscesses, and left navicular osteitis. The mycotic aneurysms were surgically removed, and vascular reconstruction by arterial allograft bypass was performed. *S. apiospermum* was identified in cultured perioperative samples; histologic analysis revealed vascular invasion, particularly in the tunica media (Figure 3, panels C, D). The patient was successfully treated with voriconazole and caspofungin for 18 months; PET/CT showed no signs of relapse 1 year after stopping antifungal therapy.

**Figure 4:**
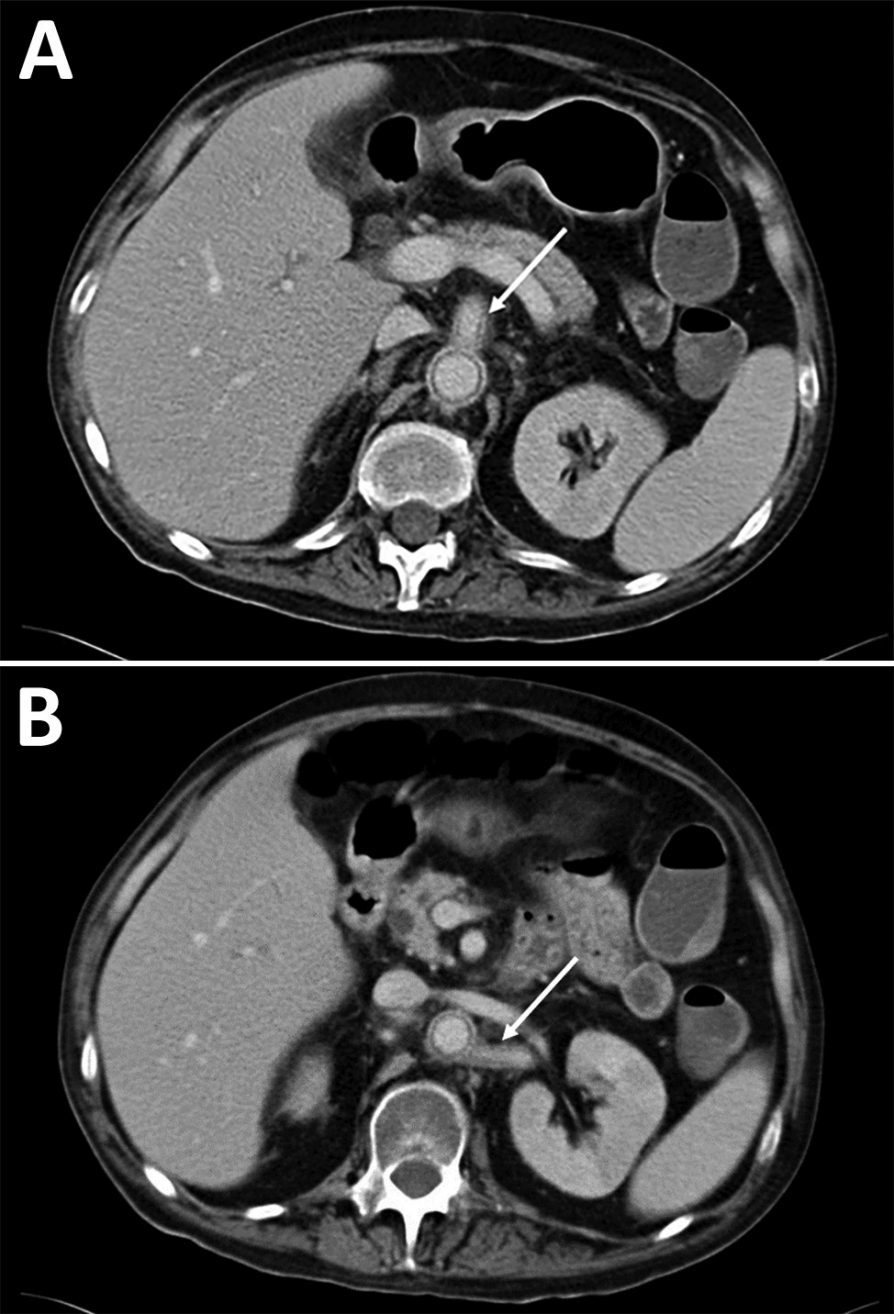
Abdominal computed tomography scans for case-patient 5 showing aortitis in study of unexpected vascular locations of *Scedosporium* spp. and *Lomentospora prolificans* fungal infections, France. A) Arrow indicates abdominal aorta and superior mesenteric artery thickening and perivascular contrast. B) Arrow indicates abdominal aorta and left renal artery thickening and perivascular contrast. Data are from the Scedosporiosis/lomentosporiosis Observational Study (*12*).

Case-patient 5 was a 44-year-old man who had chronic myeloid leukemia and underwent allogeneic hematopoietic stem cell transplant. While he was hospitalized for chronic graft versus host disease, a febrile gingival abscess developed and was treated by dental avulsion. Abscess and blood culture samples tested positive for *L. prolificans*; voriconazole and terbinafine treatments were initiated. Although fungemia persisted during treatment, he complained about abdominal pain; CT revealed abdominal aortitis spreading to the superior mesenteric and left renal arteries manifested by vascular thickenings and perivascular contrast (Figure 4, panels A, B). Within the following month, he had intense abdominal pain and died, likely from mesenteric ischemia.

**Figure 5:**
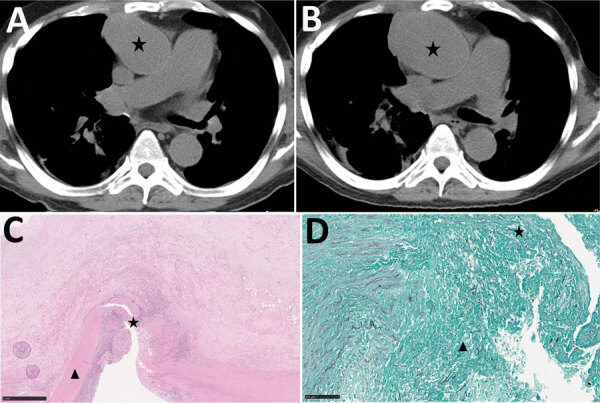
Radiologic and histopathologic analysis of thoracic aortitis in case-patient 6 in study of unexpected vascular locations of *Scedosporium* spp. and *Lomentospora prolificans* fungal infections, France. A) Thoracic computed tomography scan showing sacciform aneurysm of ascending aorta (star). B) Thoracic computed tomography scan 21 days later showing the rapidly growing aneurysm (star). C) Hematoxylin-eosin-saffron stain of thoracic aorta section. Star indicates thoracic aorta wall dissection. Triangle indicates tunica media. Scale bar indicates 1 mm. D) Grocott-Gomori methenamine silver stain of thoracic aorta section. Star indicates septate fungal hyphae invading the thoracic aorta tunica media. Triangle indicates thrombus containing septate fungal hyphae. Scale bar indicates 100 μm. Data are from the Scedosporiosis/lomentosporiosis Observational Study (*12*).

Case-patient 6 was a 56-year-old man who had diabetes and a lung transplant because of idiopathic chronic pulmonary fibrosis. A thoracotomy scar abscess developed rapidly after his transplantation surgery and was treated by surgical abscess drainage. *S. apiospermum* was documented in a perioperative specimen; therefore, he was treated with voriconazole. Four months later, while still undergoing antifungal treatment, he complained about a nonfebrile thoracic pain; CT revealed a rapidly growing ascending thoracic aortic aneurysm (Figure 5, panels A, B). Surgical aneurysm resection and prosthetic aortic replacement were performed, and he was treated with posaconazole and caspofungin. His perioperative tissue samples confirmed vascular fungal invasion (Figure 5, panels C, D), and cultures tested positive for *S. apiospermum*. After an initial clinical improvement, he died 3 months later; *S. apiospermum* fungemia had developed along with endocarditis complicated by massive ischemic stroke and mycotic aneurysm of the aortic prosthesis anastomosis (Appendix Figure 1). At the time of his death, he also had mycotic intrahepatic and splenic aneurysms.

Case-patient 7 was a 43-year-old woman receiving first-line chemotherapy for acute myeloid leukemia. Four days after chemotherapy initiation, febrile nodular cutaneous lesions developed that persisted despite treatment with wide-spectrum antimicrobial drugs and intravenous liposomal amphotericin B. A blood culture tested positive for *L. prolificans*, and antifungal therapy was switched to voriconazole. She died a few hours later because of refractory sepsis and acute cardiac failure. Postmortem examination revealed massive multiorgan filamentous invasion within blood vessel walls and lumens, particularly in the abdominal aorta, and within the myocardium.

### Cohort of SOS and Published Cases

We classified the 7 non-CNS vascular infection cases identified from the SOS as 1 aortitis, 2 peripheral arteritis, and 4 mixed aortitis and peripheral arteritis cases. We identified 26 articles from the published literature search as potential reports of non-CNS vascular scedosporiosis/lomentosporiosis (Appendix Figure 2). We excluded 13 reports, 1 about CNS mycotic aneurysms (n = 11), 1 about ventricular assist device thrombosis (n = 1), and 1 without available full text (n = 1). Thirteen case-reports from the literature search, including 9 aortitis and 4 peripheral arteritis cases, were eligible for analysis (*20*–*32*).

All 20 cases were proven IS (Appendix Table). Vascular infection was confirmed by either histology or vascular sample cultures in 15 cases. For the 5 remaining cases, vascular samples were not available. For those, diagnoses were made on the basis of radiologic imaging compatible with vascular infection and proven IS at another site. All mycologic documentations were obtained from cultures of normally sterile sites.

### Host Factors/Underlying Diseases

All but 1 patient had >1 risk factor for IS (Table 1). The main underlying condition was solid organ transplant (10/20 [50%]), including lung (n = 5), kidney (n = 3), heart (n = 1), liver/kidney (n = 1) transplants, followed by hematologic malignancy (3/20 [15%]) and solid cancer (1/20 [5%]). IS occurred with a median delay of 165 (interquartile range 25.8–271.3) days after transplantation. Five (25%) cases were associated with trauma or near-drowning, 3 (15%) cases were breakthrough infections, and 2 (10%) patients had prior *Scedosporium* spp. or *L. prolificans* colonization.

**Table 1:** General patient characteristics in study of unexpected vascular locations of *Scedosporium* spp. and *Lomentospora prolificans* fungal infections, France*

Variables	SOS, n = 7	Literature, n = 13	Total, n = 20
Type of vascular infection			
Aortitis	5 (71)	9 (69)	14 (70)
Peripheral arteritis	6 (86)	4 (31)	10 (50)
Age, y (IQR)	44.0 (37.1–51.5)	55.0 (44.0–64.0)	52.5 (42.0–57.4)
Sex			
M	4 (57)	6 (46)	10 (50)
F	3 (43)	7 (54)	10 (50)
Main underlying disease/condition			
Malignancy	2 (29)	2 (15)	4 (20)
Including hemopathy	2 (29)	1 (8)	3 (15)
Including solid neoplasia	0	1 (8)	1 (5)
Solid organ transplant	3 (43)	7 (54)	10 (50)
Trauma	2 (29)	2 (15)	4 (20)
Near drowning	0	1 (8)	1 (5)
No risk factor	0	1 (8)	1 (5)
Neutropenia	1 (14)	1 (8)	2 (10)
Prior colonization	0	2 (15)	2 (10)
Breakthrough infections	1 (14)	2 (15)	3 (15)
Fungus identification			
* S. apiospermum*	4 (57)	8 (62)	12 (60)
* L. prolificans*	2 (29)	5 (39)	7 (35)
*S. apiospermum* and *L. prolificans*	1 (14)	0	1 (5)
Disseminated infection	7 (100)	8 (62)	15 (75)
Fungemia†	4/17 (57)	4/11 (36)	8/18 (44)

*Values are no. (%) except as indicated. Patients had non–central nervous system vascular infections caused by *Scedosporium* spp. or *Lomentospora prolificans*; cases were obtained from the SOS and from published literature. IQR, interquartile range; SOS, Scedosporiosis/lomentosporiosis Observational Study. †Blood culture data were not available from published literature for 2 patients. Denominators indicate the total number of patients evaluated.

### Mycologic and Clinical Manifestations

*S. apiospermum* complex species were identified in 12 (60%) cases, and *L. prolificans* was identified in 7 (35%) cases. Co-infection with *S. apiospermum* and *L. prolificans* occurred in 1 patient. Fungemia was reported in 8 (44%) cases, more frequently among *L. prolificans* infections (4/5 [80%]) than *S. apiospermum* complex infections (4/12 [33%]). Blood samples were not cultured for 2 patients with *L. prolificans* infection. Disseminated infections occurred in 15 (75%) cases, among which 13 had >2 noncontiguous infectious sites.

IS manifested as fever in 9/15 (60%) cases (Table 2). Patients with aortitis frequently (7/14 [50%]) had thoracic or abdominal pain. Vascular impairment was not observed at the time of IS diagnosis in 10/20 (50%) cases. However, impairment was frequently diagnosed months or years after completion of antifungal treatment for nonvascular scedosporiosis/lomentosporiosis (9/20 [45%]) but also while patients were still undergoing antifungal treatment (1/20 [5%]). The median delay between completion of antifungal treatment and diagnosis was 3 (interquartile range 1–13) months (4 years maximum). Moreover, vascular impairment was associated with >1 other localization in 18/20 (90%) cases, mostly in osteoarticular (10/20 [50%]) and pulmonary (9/20 [45%]) locations; 5/10 (50%) osteoarticular localizations were spondylodiscitis. Most (7/10 [70%]) osteoarticular localizations were contiguous with the vascular infection site, which included 3 of 5 spondylodiscitis (2 abdominal and 1 thoracic aortitis) and 4 of 5 peripheral osteoarticular localizations (1 femoral arteritis and hip arthritis, 1 iliac arteritis and sacroiliac arthritis, 1 sternal osteomyelitis and thoracic aortitis, and 1 subclavian arteritis and sternoclavicular arthritis).

**Table 2:** Clinical and radiologic characteristics of patients in study of unexpected vascular locations of *Scedosporium* spp. and *Lomentospora prolificans* fungal infections, France*

Variables		SOS, n = 7	Literature, n = 13	Total, n = 20
Clinical manifestations at diagnosis				
Fever		6/7 (86)	3/8 (38)	9/15 (60)
Sepsis		1/7 (14)	2/13 (15)	3/20 (15)
Thoracic or abdominal pain among aortitis		2/5 (40)	5/9 (56)	7/14 (50)
Local inflammatory sign among peripheral arteritis		0	2/4 (50)	2/10 (20)
Vascular involvement at scedosporiosis/lomentosporiosis diagnosis	5/7 (71)		5/13 (39)	10/20 (50)
Other infection locations				
Pulmonary		4/7 (57)	5/13 (39)	9/20 (45)
Central nervous system		2/7 (29)	3/13 (23)	5/20 (25)
Cutaneous		2/7 (29)	2/13 (15)	4/20 (20)
Osteoarticular		3/7 (43)	7/13 (54)	10/20 (50)
Including spondylodiscitis		1/3 (33)	4/7 (57)	5/10 (50)
Including contiguous localizations		1/3 (33)	6/7 (86)	7/10 (70)
Endocarditis		2/7 (29)	2/13 (15)	4/20 (20)
Localization of vascular infection				
Aortitis		5/7 (71)	9/13 (69)	14/20 (70)
Including thoracic aortitis		1/5 (20)	4/8 (50)	5/13 (39)
Including abdominal aortitis		4/5 (80)	4/8 (50)	8/13 (62)
Peripheral arteritis		6/7 (86)	4/13 (31)	10/20 (50)
Including iliac or femoral arteritis		3/6 (50)	1/4 (25)	4/10 (40)
Type of aortitis vascular impairment				
Aneurysm		1/4 (25)	7/7 (100)	8/11 (73)
With rupture		1/1 (100)	2/7 (29)	3/8 (38)
Perivascular abscess		1/4 (25)	0	1/11 (9)
Isolated vascular thickening		2/4 (50)	0	2/11 (18)
Type of peripheral arteritis vascular impairment				
Aneurysm		3/6 (50)	3/4 (75)	6/10 (60)
With rupture		0	1/3 (33)	1/6 (17)
Perivascular abscess		1/6 (17)	0	1/10 (10)
Septic thrombosis		2/6 (33)	1/4 (25)	3/10 (30)
Isolated vascular thickening		1/6 (17)	0	1/10 (10)
Vascular hypermetabolism on PET/CT		3/3 (100)	3/3 (100)	6/6 (100)

*Values are no./total no. analyzed (%). Denominators are different in some cases because of missing data. Patients had non–central nervous system vascular infections caused by *Scedosporium* spp. or *Lomentospora prolificans*; cases were obtained from the SOS and published literature. PET/CT, positron emission tomography/computed tomography; SOS, Scedosporiosis/lomentosporiosis Observational Study.

### Vascular Impairment Type

Aortitis affected the abdominal aorta (8/13 [62%]) more frequently than the thoracic aorta (5/13 [39%]) (Table 2). The location of aortitis was not mentioned for 1 case. Aortitis was mostly identified by the presence of an aneurysmal lesion (8/11 [73%]). Indeed, aneurysmal lesions were noted for all thoracic aortitis and 3 of 6 abdominal aortitis cases. Abdominal aortitis with arterial wall thickening without aneurysms were described for SOS case-patients 1, 3, and 5. The type of imaging anomalies was unknown for 3 patients who had postmortem aortitis diagnoses.

Peripheral arteritis localizations were diverse, affecting the iliac or femoral arteries (4/10 [40%]) and other arteries, such as hepatic (n = 2), pulmonary (n = 1), subclavian (n = 1), renal and mesenteric (n = 1), or arteriovenous fistula (n = 1) arteries. Peripheral arteritis was mainly revealed by the presence of aneurysmal lesions (6/10 [60%]); septic thrombosis was described in 3 cases.

PET/CT consistently showed an elective hypermetabolism of the vascular wall in 3 aneurysmal aortitis, 2 nonaneurysmal aortitis, and 1 peripheral mycotic aneurysm cases. All positive PET/CT scans were associated with concordant CT or magnetic resonance imaging angiography. PET/CT follow-up was available for 3 of 6 patients, and a clear regression of hypermetabolism during IS treatment was reported in all 3 cases, including 1 normalization at the end of treatment.

### Histopathologic Lesions

Histopathologic analyses were available for 11/20 patients (8 perioperative vascular samples and 3 autopsies) and showed an invasion of the arterial wall by fungal septate hyphae in 10 of 11 samples. Arterial wall necrosis was reported in 6 cases and endoluminal thrombi caused by fungi were reported in 4 cases. Only 1 case showed an arterial granuloma lesion, and 2 cases had arterial wall abscesses. Although rarely specified, fungal invasion affected the tunica media (n = 3) or tunica adventitia (n = 3).

### Treatment and Outcomes

After microbiologic identification, voriconazole was the first-line antifungal drug prescribed to all patients (Table 3). A combination of 2 antifungal drugs was frequently (10/16 [63%]) used, mostly voriconazole combined with terbinafine or caspofungin. Four patients did not receive antifungal treatment because the IS diagnosis was made postmortem. Radical surgery was performed in 8 (50%) of 16 patients with an antemortem IS diagnosis, which consisted of 3 biologic vascular reconstructions (2 arterial allografts and 1 arterial homograft), 3 prosthetic vascular reconstructions (Dacron grafts in all cases), and 2 cases without vascular reconstruction. Signs of infection relapse at the vascular sites were reported in the 3 prosthetic vascular reconstruction cases (pseudoaneurysm appearance or prosthetic graft thrombosis).

**Table 3:** Patient treatment and outcomes in study of unexpected vascular locations of *Scedosporium* spp. and *Lomentospora prolificans* fungal infections, France*

Variables	SOS, n = 7	Literature, n = 13	Total, n = 20
Radical surgery	2/7 (29)	6/13 (46)	8/20 (40)
First-line antifungal treatment after microbiologic identification			
Monotherapy	1/7 (14)	3/9 (33)	4/16 (25)
Combination therapy	5/7 (71)	5/9 (56)	10/16 (63)
Multitherapy, >3 antifungal agents	1/7 (14)	1/9 (11)	2/16 (13)
Voriconazole	7/7 (100)	9/9 (100)	16/16 (100)
Terbinafine	2/7 (29)	6/9 (67)	8/16 (50)
Caspofungin	5/7 (71)	1/9 (11)	6/16 (38)
Overall IFD-related deaths	5/7 (71)	7/11 (64)	12/18 (67)
Among patients with fungemia	4/4 (100)	3/3 (100)	7/7 (100)
Among patients with aortitis whether or not associated with arteritis	4/5 (80)	7/9 (78)	11/14 (79)
Among patients with arteritis only	1/2 (50)	0/2 (0)	1/4 (25)
Among patients treated with radical surgery and antifungal	1/2 (50)	1/4 (25)	2/6 (33)
Among patients treated with antifungal only	4/5 (80)	3/4 (75)	7/9 (78)
1-month IFD-related deaths	2/7 (29)	5/11 (46)	7/18 (39)

*Values are no./total no. analyzed (%). Denominators are different in some cases because of missing data. Patients had non–central nervous system vascular infections caused by *Scedosporium* spp. or *Lomentospora prolificans*; cases were obtained from the SOS and published literature. IFD, invasive fungal disease; SOS, Scedosporiosis/lomentosporiosis Observational Study.

The IFD-related death rate was 67% (12/18) and 100% (7/7) when fungemia was present. Three IFD-related deaths were attributed to a direct complication of the vascular infection (mesenteric ischemia complicating abdominal aortitis in all cases), but the cause of death was missing for 4 patients. The death rate was higher (11/14 [79%]) for patients with aortitis (whether or not associated with peripheral arteritis) than isolated peripheral arteritis (1/4 [25%]). Patients treated with only antifungal drugs had a higher (7/9 [78%]) death rate than those who underwent radical surgery combined with antifungal drug therapy ( 2/6 [33%]).

## Discussion

We describe non-CNS vascular infections caused by *Scedosporium* spp. and *L. prolificans*, highlighting an underreported localization of clinical significance that affects the management of such infections. Solid organ transplant was the main host risk factor; however, several cases occurred in immunocompetent patients, particularly those who had cutaneous trauma. Vascular invasion was almost always associated with other infectious localizations, such as contiguous osteoarticular localizations. The main radiologic manifestation of infection was a vascular aneurysm, which was observed in all thoracic aortitis and most peripheral arteritis cases. Isolated vascular wall thickening was frequently described for abdominal aortitis and contiguous aortic branches, constituting overlap between abdominal aortitis and iliofemoral arteritis.

The SOS found that 24% of disseminated scedosporiosis/lomentosporiosis cases involved vascular infections (*5*–*11*,*13*–*17*). No non-CNS vascular infections were reported among 273 IS cases according to the Fungiscope registry (*5*), which gathers rare IFD data from countries around the world. Only 2 cases of mycotic aneurysms were reported among 80 scedosporiosis/lomentosporiosis cases in transplant patients, of which 40 were disseminated (*33*). One possible explanation for this underreporting might be that vascular impairment is rarely at the forefront of IS diagnostic considerations and might not be systematically tracked by imaging checkups or might be insufficiently reported in disseminated cases. All cases from published literature were reported since 2000, which could be partly explained by recent improvements in imaging access. In addition, vascular impairment was diagnosed in many (45%) cases after patients completed antifungal treatment for another IS localization, suggesting that undiagnosed vascular infections might lead to relapse because of insufficient treatment. We found that 50% of IS cases were associated with osteoarticular localization, whereas that localization was usually described in only 18%–26% IS and 14%–17% of disseminated scedosporiosis/lomentosporiosis cases (*5*,*12*,*17*). This finding suggests a potential association between vascular and osteoarticular localizations, especially because most of those were contiguous. In particular, we highlighted a frequent association of peripheral arteritis with aortitis resulting from spondylodiscitis and peripheral osteoarthritis. Finally, we reported high death rates, consistent with previous estimations for overall IS (*5*,*7*).

We focused on non-CNS vascular infections because cerebral mycotic aneurysms caused by *Scedosporium* spp. have already been specifically addressed for near-drowning situations (*34*). The ability of molds to invade vessels is well known; angioinvasion is a key pathogenic characteristic of invasive aspergillosis and mucormycosis. Several reports have described cerebral and pulmonary mycotic aneurysms located contiguously or distant to invasive aspergillosis or mucormycosis (*35*–*39*). However, few studies have reported thoracic aortitis caused by *Aspergillus* spp., which are frequently associated with endocarditis (*40*–*42*).

The strength of this work is that it confirms arterial wall invasion by *Scedosporium* or *Lomentospora* hyphae; histopathologic analyses were available for most cases. Nevertheless, IFD reporting is voluntary in France, and the National Reference Center might not have gathered data for all IFD cases in France during the study period. Moreover, we could not provide microbiologic identification at the species level for *S. apiospermum* complex species because genotypic analysis was conducted in only 4 of 13 cases reported in published literature, and several diagnoses were made before the last taxonomy classification update. We could not analyze the median duration of antifungal therapy because all but 3 patients were still undergoing treatment at last contact. Also, anticoagulant and antiplatelet drug management could not be assessed because of missing data. We were unable to evaluate the effects of vascular impairment on scedosporiosis/lomentosporiosis prognosis because assessing the exact circumstances of death was difficult in many cases; however, >3 of the 12 IFD-related deaths seemed to be related to a vascular complication. Finally, we observed that patients who underwent radical surgery seemed to have better prognoses. However, the retrospective study design, small number of cases, and patient population heterogeneity do not permit assessment of the best curative treatment, leading us to interpret the results for radical surgery with caution.

In conclusion, arterial wall infection is observed in 24% of patients with disseminated *Scedosporium*/*Lomentospora* infections and is mainly responsible for arterial aneurysms either by contiguous contamination or systemic dissemination. Our case series and literature review suggest that *Scedosporium* spp. and *L. prolificans* arterial infections might be latent for months after IS diagnosis and treatment, and ad hoc vascular imaging is justified in cases of osteoarticular localization or dissemination. Systematic screening for vascular involvement of *Scedosporium*/*Lomentospora* infections, including aortitis and peripheral arteritis, is needed to improve therapeutic strategies and prognosis.

Appendix Additional information for deciphering unexpected vascular locations of *Scedosporium* spp. and *Lomentospora prolificans* fungal infections, France.

## References

[1] Ramirez-Garcia A, Pellon A, Rementeria A, Buldain I, Barreto-Bergter E, Rollin-Pinheiro R, et al. *Scedosporium* and *Lomentospora*: an updated overview of underrated opportunists. Med Mycol. 2018;56(suppl_1):102–25. 10.1093/mmy/myx113 29538735

[2] Miceli MH, Lee SA. Emerging moulds: epidemiological trends and antifungal resistance. Mycoses. 2011;54:e666–78. 10.1111/j.1439-0507.2011.02032.x 21672045

[3] Lackner M, de Hoog GS, Yang L, Ferreira Moreno L, Ahmed SA, Andreas F, et al. Proposed nomenclature for *Pseudallescheria, Scedosporium* and related genera. Fungal Divers. 2014;67:1–10. 10.1007/s13225-014-0295-4

[4] Blez D, Bronnimann D, Rammaert B, Zeller V, Delhaes L, Hustache L, et al. Invasive bone and joint infections from the French Scedosporiosis/lomentosporiosis Observational Study (SOS) cohort: no mortality with long-term antifungal treatment and surgery. Med Mycol. 2023;61:myad023. 10.1093/mmy/myad023 36813259

[5] Seidel D, Meißner A, Lackner M, Piepenbrock E, Salmanton-García J, Stecher M, et al. Prognostic factors in 264 adults with invasive Scedosporium spp. and Lomentospora prolificans infection reported in the literature and FungiScope^®^. Crit Rev Microbiol. 2019;45:1–21. 10.1080/1040841X.2018.1514366 30628529

[6] Jenks JD, Seidel D, Cornely OA, Chen S, van Hal S, Kauffman C, et al. Clinical characteristics and outcomes of invasive Lomentospora prolificans infections: Analysis of patients in the FungiScope^®^ registry. Mycoses. 2020;63:437–42. 10.1111/myc.13067 32080902

[7] Álvarez-Uría A, Guinea JV, Escribano P, Gómez-Castellá J, Valerio M, Galar A, et al. Invasive *Scedosporium* and *Lomentosora* infections in the era of antifungal prophylaxis: A 20-year experience from a single centre in Spain. Mycoses. 2020;63:1195–202. 10.1111/myc.13154 32749009

[8] Lamaris GA, Chamilos G, Lewis RE, Safdar A, Raad II, Kontoyiannis DP. *Scedosporium* infection in a tertiary care cancer center: a review of 25 cases from 1989-2006. Clin Infect Dis. 2006;43:1580–4. 10.1086/509579 17109292

[9] Troke P, Aguirrebengoa K, Arteaga C, Ellis D, Heath CH, Lutsar I, et al.; Global Scedosporium Study Group. Treatment of scedosporiosis with voriconazole: clinical experience with 107 patients. Antimicrob Agents Chemother. 2008;52:1743–50. 10.1128/AAC.01388-07 18212110 PMC2346616

[10] Idigoras P, Pérez-Trallero E, Piñeiro L, Larruskain J, López-Lopategui MC, Rodríguez N, et al. Disseminated infection and colonization by *Scedosporium prolificans*: a review of 18 cases, 1990-1999. Clin Infect Dis. 2001;32:E158–65. 10.1086/320521 11340550

[11] Heath CH, Slavin MA, Sorrell TC, Handke R, Harun A, Phillips M, et al.; Australian Scedosporium Study Group. Population-based surveillance for scedosporiosis in Australia: epidemiology, disease manifestations and emergence of Scedosporium aurantiacum infection. Clin Microbiol Infect. 2009;15:689–93. 10.1111/j.1469-0691.2009.02802.x 19549223

[12] Bronnimann D, Garcia-Hermoso D, Dromer F, Lanternier F, Maulin L, Leprince Y, et al.; French Mycoses Study Group; Characterization of the isolates at the NRCMA. Scedosporiosis/lomentosporiosis observational study (SOS): Clinical significance of Scedosporium species identification. Med Mycol. 2021;59:486–97. 10.1093/mmy/myaa086 33037432

[13] Berenguer J, Rodríguez-Tudela JL, Richard C, Alvarez M, Sanz MA, Gaztelurrutia L, et al.; Scedosporium Prolificans Spanish Study Group. Deep infections caused by Scedosporium prolificans. A report on 16 cases in Spain and a review of the literature. Medicine (Baltimore). 1997;76:256–65. 10.1097/00005792-199707000-00004 9279332

[14] Tintelnot K, Just-Nübling G, Horré R, Graf B, Sobottka I, Seibold M, et al. A review of German *Scedosporium prolificans* cases from 1993 to 2007. Med Mycol. 2009;47:351–8. 10.1080/13693780802627440 19301173

[15] Seidel D, Hassler A, Salmanton-García J, Koehler P, Mellinghoff SC, Carlesse F, et al. Invasive *Scedosporium* spp. and *Lomentospora prolificans* infections in pediatric patients: Analysis of 55 cases from FungiScope® and the literature. Int J Infect Dis. 2020;92:114–22. 10.1016/j.ijid.2019.12.017 31863876

[16] Johnson LS, Shields RK, Clancy CJ. Epidemiology, clinical manifestations, and outcomes of *Scedosporium* infections among solid organ transplant recipients. Transpl Infect Dis. 2014;16:578–87. 10.1111/tid.12244 24962102

[17] Rodriguez-Tudela JL, Berenguer J, Guarro J, Kantarcioglu AS, Horre R, de Hoog GS, et al. Epidemiology and outcome of *Scedosporium prolificans* infection, a review of 162 cases. Med Mycol. 2009;47:359–70. 10.1080/13693780802524506 19031336

[18] Carrer M, Vignals C, Berard X, Caradu C, Battut AS, Stenson K, et al. Retrospective multicentric study comparing infectious and noninfectious aortitis. Clin Infect Dis. 2023;76:e1369–78. 10.1093/cid/ciac560 35792621

[19] Donnelly JP, Chen SC, Kauffman CA, Steinbach WJ, Baddley JW, Verweij PE, et al. Revision and Update of the Consensus Definitions of Invasive Fungal Disease From the European Organization for Research and Treatment of Cancer and the Mycoses Study Group Education and Research Consortium. Clin Infect Dis. 2020;71:1367–76. 10.1093/cid/ciz1008 31802125 PMC7486838

[20] Ong A, Blyth CC, Bency R, Vicaretti M, Harun A, Meyer W, et al. Fatal mycotic aneurysms due to *Scedosporium* and *Pseudallescheria* infection. J Clin Microbiol. 2011;49:2067–71. 10.1128/JCM.02615-10 21430108 PMC3122655

[21] Howden BP, Slavin MA, Schwarer AP, Mijch AM. Successful control of disseminated *Scedosporium prolificans* infection with a combination of voriconazole and terbinafine. Eur J Clin Microbiol Infect Dis. 2003;22:111–3. 10.1007/s10096-002-0877-z 12627286

[22] Ortmann C, Wüllenweber J, Brinkmann B, Fracasso T. Fatal mycotic aneurysm caused by *Pseudallescheria boydii* after near drowning. Int J Legal Med. 2010;124:243–7. 10.1007/s00414-009-0336-9 19294403

[23] Malinowski MJ, Halandras P. Arterial reconstruction for atypical mycotic aneurysms. Vasc Endovascular Surg. 2013;47:45–7. 10.1177/1538574412462636 23047819

[24] Centellas Pérez FJ, Martínez Antolinos C, Piqueras Sánchez S, Lorenzo González I, Llamas Fuentes F, Gómez Roldán C. [*Scedosporium apiospermum* infection in a kidney transplant recipient] [in Spanish]. Rev Iberoam Micol. 2019;36:48–50. 30833046 10.1016/j.riam.2018.10.005

[25] Klinken EM, Stevenson BR, Kwok CHR, Hockley JA, Lucas M. Diffuse inflammatory aneurysmal aortitis secondary to *Scedosporium apiospermum* complex in an immunocompetent individual. Pathology. 2019;51:316–8. 10.1016/j.pathol.2018.10.021 30819537

[26] Blasco-Lucas A, Reyes-Juárez JL, Nazarena Pizzi M, Permanyer E, Evangelista A, Galiñanes M. Aortic arch mycotic aneurysm due to *Scedosporium apiospermum* reconstructed with homografts. Ann Thorac Surg. 2015;99:2218–20. 10.1016/j.athoracsur.2014.08.067 26046885

[27] Thomson S, Alibhai K, Winkelaar G, Lien D, Halloran K, Kapasi A, et al. Case report of vertebral osteomyelitis and mycotic abdominal aortic aneurysm caused by *Scedosporium apiospermum* in a lung transplant patient with cystic fibrosis. Transplant Proc. 2015;47:204–9. 10.1016/j.transproceed.2014.07.072 25645805

[28] Sayah DM, Schwartz BS, Kukreja J, Singer JP, Golden JA, Leard LE. *Scedosporium prolificans* pericarditis and mycotic aortic aneurysm in a lung transplant recipient receiving voriconazole prophylaxis. Transpl Infect Dis. 2013;15:E70–4. 10.1111/tid.12056 23387799

[29] Wakabayashi Y, Okugawa S, Tatsuno K, Ikeda M, Misawa Y, Koyano S, et al. *Scedosporium prolificans* endocarditis: case report and literature review. Intern Med. 2016;55:79–82. 10.2169/internalmedicine.55.5592 26726091

[30] Valerio M, Vásquez V, Álvarez-Uria A, Zatarain-Nicolás E, Pavone P, Martínez-Jiménez MDC, et al. Disseminated lomentosporiosis in a heart transplant recipient: Case report and review of the literature. Transpl Infect Dis. 2021;23:e13574. 10.1111/tid.13574 33527651

[31] Holmes NE, Trevillyan JM, Kidd SE, Leong TYM. Locally extensive angio-invasive *Scedosporium prolificans* infection following resection for squamous cell lung carcinoma. Med Mycol Case Rep. 2013;2:98–102. 10.1016/j.mmcr.2013.04.001 24432228 PMC3885965

[32] Campa-Thompson MM, West JA, Guileyardo JM, Spak CW, Sloan LM, Beal SG. Clinical and morphologic findings in disseminated *Scedosporium apiospermum* infections in immunocompromised patients. Proc Bayl Univ Med Cent. 2014;27:253–6. 10.1080/08998280.2014.11929129 24982580 PMC4059584

[33] Husain S, Muñoz P, Forrest G, Alexander BD, Somani J, Brennan K, et al. Infections due to *Scedosporium apiospermum* and *Scedosporium prolificans* in transplant recipients: clinical characteristics and impact of antifungal agent therapy on outcome. Clin Infect Dis. 2005;40:89–99. 10.1086/426445 15614697

[34] Katragkou A, Dotis J, Kotsiou M, Tamiolaki M, Roilides E. *Scedosporium apiospermum* infection after near-drowning. Mycoses. 2007;50:412–21. 10.1111/j.1439-0507.2007.01388.x 17714363

[35] Hurst RW, Judkins A, Bolger W, Chu A, Loevner LA. Mycotic aneurysm and cerebral infarction resulting from fungal sinusitis: imaging and pathologic correlation. AJNR Am J Neuroradiol. 2001;22:858–63. 11337328 PMC8174942

[36] Kasliwal MK, Reddy VSK, Sinha S, Sharma BS, Das P, Suri V. Bilateral anterior cerebral artery aneurysm due to mucormycosis. J Clin Neurosci. 2009;16:156–9. 10.1016/j.jocn.2008.04.019 19013802

[37] Sundaram C, Goel D, Uppin SG, Seethajayalakshmi S, Borgohain R. Intracranial mycotic aneurysm due to *Aspergillus* species. J Clin Neurosci. 2007;14:882–6. 10.1016/j.jocn.2006.05.014 17660058

[38] Coffey MJ, Fantone J III, Stirling MC, Lynch JP III. Pseudoaneurysm of pulmonary artery in mucormycosis. Radiographic characteristics and management. Am Rev Respir Dis. 1992;145:1487–90. 10.1164/ajrccm/145.6.1487 1596023

[39] Lopez-Pastorini A, Koryllos A, Brockmann M, Windisch W, Stoelben E. Pseudoaneurysm of the pulmonary artery with massive haemoptysis due to an invasive pulmonary mucormycosis. Thorax. 2016;71:199–200. 10.1136/thoraxjnl-2015-207713 26385775

[40] Silva ME, Malogolowkin MH, Hall TR, Sadeghi AM, Krogstad P. Mycotic aneurysm of the thoracic aorta due to *Aspergillus terreus*: case report and review. Clin Infect Dis. 2000;31:1144–8. 10.1086/317467 11073743

[41] Rose HD, Stuart JL. Mycotic aneurysm of the thoracic aorta caused by A*spergillus fumigatus.* Chest. 1976;70:81–4. 10.1378/chest.70.1.81 776547

[42] Sanchez-Recalde A, Maté I, Merino JL, Simon RS, Sobrino JA. Aspergillus aortitis after cardiac surgery. J Am Coll Cardiol. 2003;41:152–6. 10.1016/S0735-1097(02)02606-2 12570958

