# Supplementary material for: Deciphering Unexpected Vascular Locations of Scedosporium spp. and Lomentospora prolificans Fungal Infections, France
Source: Emerg Infect Dis. 2024 Jun;30(6):1077–87. doi: 10.3201/eid3006.231409 (PMC11138966; doi:10.3201/eid3006.231409)
Supplement: Appendix — Additional information for deciphering unexpected vascular locations of Scedosporium spp. and Lomentospora prolificans fungal infections, France. [file 23-1409-Techapp-s1.pdf]

Article DOI: https://doi.org/10.3201/eid3006.231409

EID cannot ensure accessibility for supplementary materials supplied by authors. Readers who have difficulty accessing supplementary content should contact the authors for assistance.

Deciphering Unexpected Vascular Locations of *Scedosporium* spp. and *Lomentospora prolificans* Fungal Infections, France

Appendix

Imaging Criteria

Imaging criteria for aortic/arterial wall structural abnormalities used to diagnose aortitis/arteritis consist of computed tomography or magnetic resonance imaging showing either aortic or arterial wall thickening, perivascular and soft tissue contrast uptake, false aneurysm, or rapid aneurysmal growth, or positron emission tomography-computed tomography showing tracer accumulation in a vascular segment or hypermetabolic aneurysm.

Appendix Table. Description of non-CNS vascular infection cases caused by *Scedosporium* spp. and *Lomentospora prolificans* from the Scedosporiosis/lomentosporiosis Observational Study and from published literature*

Case or reference no.†	Age, y/sex	Main underlying condition	Vascular involvement	Other infected sites	Microbiologic documentation	Duration of antifungal management, d‡	Radical surgery	Outcome at follow-up, d§
Case 1	52/M	SOT, liver/kidney	Primitive iliac arteritis, aortic bifurcation, aortitis	Adjacent (sacroiliitis, L5-S1 spondylodiscitis) and distant osteoarticular	*S. apiospermum* in joint fluid	VRC + CAS + TRB, n = 53	No	Death related to IFD, n = 53
Case 2	31/F	Intravenous drug user, road accident in muddy water	Pulmonary arteritis	Tricuspid endocarditis, cerebral abscess, pulmonary embolisms	*S. apiospermum* in blood culture, sputum	VRC + CAS, n = 930	No	Death related to IFD, n = 930
Case 3	32/M	Hemophagocytic syndrome because of acute cocaine intoxication	Abdominal aortitis, primitive iliac arteritis	Multiple distant osteoarticular, pneumonia	*S. apiospermum* in joint fluid, *L. prolificans* in bone biopsy	VRC, then VRC + TRB + MLT, n = 1,095	No	Survival, n = 1,460
Case 4	51/F	SOT, kidney	Common femoral arteritis	Adjacent soft tissue and distant osteoarticular	*S. apiospermum* in iliofemoral artery, soft tissue abscess	VRC + CAS, n = 540	Yes	Death unrelated to IFD, n = 750
Case 5	44/M	HSCT and chronic GVHD	Abdominal aortitis, left renal arteritis, superior mesenteric arteritis	Gingival abscess	*L. prolificans* in blood culture, gingival abscess	VRC + TRB, n = 38	No	Death related to IFD, n = 38
Case 6	56/M	SOT, lung	Thoracic aortitis, hepatosplenic arteritis	Thoracotomy scar abscess, pneumonia, aortic endocarditis, CNS embolisms	*S. apiospermum* in blood culture, aorta	VRC + CAS, then PSC + CAS, n = 126	Yes	Death related to IFD, n = 126
Case 7	43/F	AML	Abdominal aortitis	Skin, lung, myocardium, kidneys, stomach, pancreas	L. prolificans in blood culture	VRC + CAS, n = 1	No	Death related to IFD, n = 3
(*20*)	55/M	SOT, kidney	Abdominal aortitis	Contiguous spondylodiscitis	*S. apiospermum* in aorta, paravertebral abscess	VRC, NA	Yes	Death related to IFD, n = 120
(*21*)	53/F	Myeloma	Hepatic arteritis	Distant spondylodiscitis	*L. prolificans* in hepatic aneurysm, bone biopsy	VRC + TRB, n = 450	Yes	Survival, n = 450
(*22*)	53/F	Immunocompetent, drowning	Abdominal aortitis	Pneumonia, CNS mycotic aneurysms	*S. apiospermum* in aorta, blood culture	None	No	Death related to IFD, n = 0
(*23*)	67/M	SOT, lung	Superficial femoral arteritis	Contiguous hip arthritis	*S. apiospermum* in femoral artery	VRC + TRB, NA	Yes	NA

Case or reference no.†	Age, y/sex	Main underlying condition	Vascular involvement	Other infected sites	Microbiologic documentation	Duration of antifungal management, d‡	Radical surgery	Outcome at follow-up, d§
(*24*)	55/F	SOT, kidney	Arteriovenous fistula infection	None	*S. apiospermum* in blood culture, arteriovenous fistula	VRC, NA	Yes	NA
(*25*)	74/M	Immunocompetent	Abdominal aortitis	None	*S. apiospermum* in aorta	None	No	Death related to IFD, n = 0
(*26*)	39/F	Intravenous drug user	Thoracic aortitis	Contiguous spondylodiscitis	*S. apiospermum* in aorta	VRC, n = 126	Yes	Survival, n = 147
(*27*)	19/F	SOT (lung)	Abdominal aortitis	Contiguous spondylodiscitis	*S. apiospermum* in aorta	VRC + TRB, NA	Yes	Survival, n = 84
(*28*)	70/F	SOT (lung)	Thoracic aortitis	Contiguous pneumonia and pericarditis	*L. prolificans* in pericardium, BL	VRC + TRB + CAS, n = 133	No	Death related to IFD, n = 133
(*29*)	64/F	Immunocompetent	Thoracic aortitis	Contiguous sternal osteomyelitis, aortic and tricuspid endocarditis	*L. prolificans* in blood culture	None	No	Death related to IFD, n = 0
(*30*)	25/M	SOT (heart)	Thoracic aortitis	Lung, heart, CNS, kidneys, liver, skin	L. prolificans in blood culture, BL	VRC + TRB, n = 3	No	Death related to IFD, n = 3
(*31*)	44/M	Lung carcinoma	Subclavian arteritis	Contiguous pleurisy, pericarditis, and sternoclavicular site	*L. prolificans* in pleural fluid	VRC + TRB, n = 390	No	Death unrelated to IFD, n = 755
(*32*)	62/M	SOT (lung)	Unprecise localization of aortitis	CNS, lung, heart, kidney, thyroid	*S. apiospermum* in pleural fluid, BL	None	No	Death related to IFD, n = 0

*AML, acute myeloid leukemia; BL, bronchoalveolar lavage; CAS, caspofungin; CNS, central nervous system; GVHD, graft versus host disease; HSCT, hematopoietic stem cell transplantation; IFD, invasive fungal disease; MLT, miltefosine; NA, not available; PSC, Posaconazole; SOT, solid organ transplant; TRB, terbinafine; VRC, voriconazole.
†Reference numbers are from the main text.
‡After microbiologic identification.
§Number of days between IFD diagnosis and death or last follow-up.

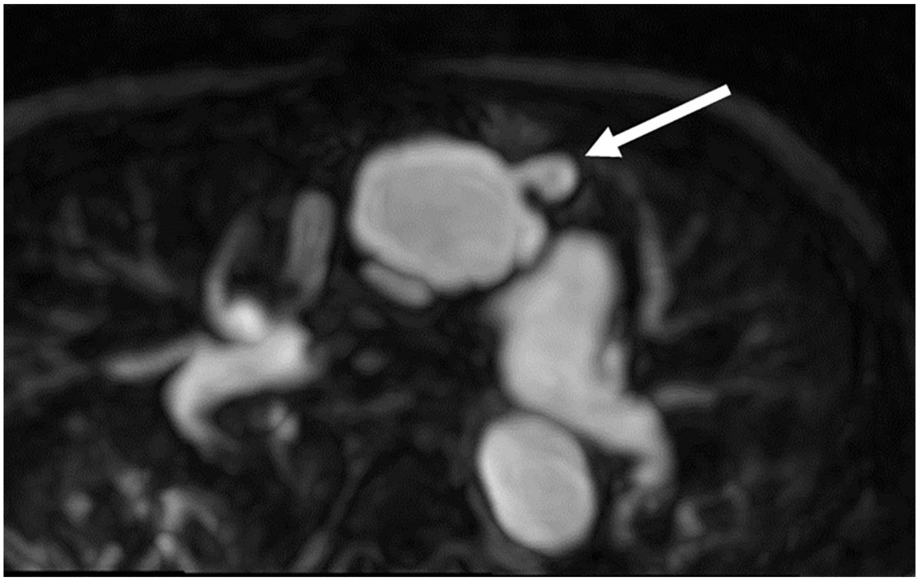

Appendix Figure 1. Thoracic magnetic resonance image of aorta prosthesis vascular infection in case-patient 6 in study of unexpected vascular locations of *Scedosporium* spp. and *Lomentospora prolificans* fungal infections, France. Arrow indicates ascending aortic prosthesis proximal to the suture’s false aneurysm. Data are from the Scedosporiosis/lomentosporiosis Observational Study.

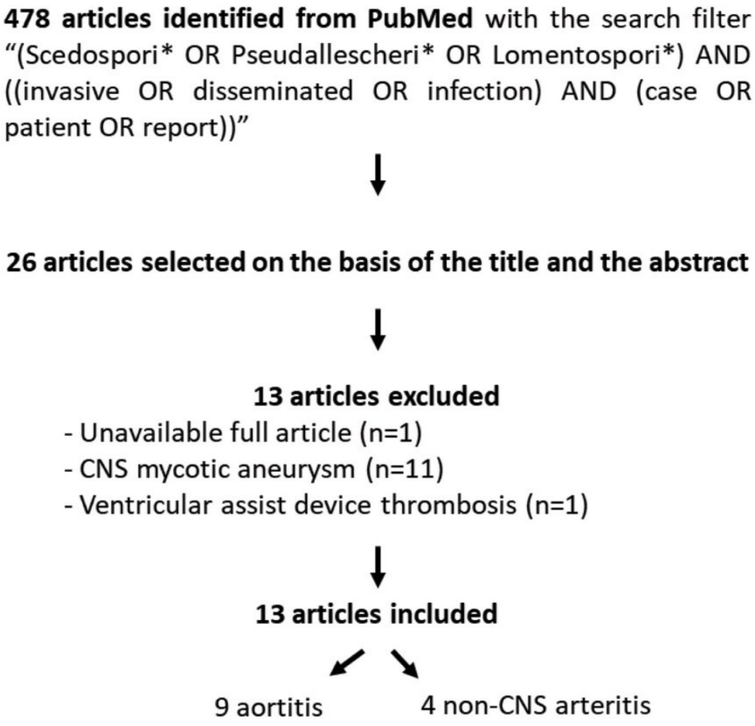

Appendix Figure 2. Flow chart showing cases selected from published reports of *Scedosporium* spp. and *Lomentospora prolificans* fungal infections. CNS, central nervous system.
